# PCSK9 inhibitors and inclisiran with or without statin therapy on incident muscle symptoms and creatine kinase: a systematic review and network meta-analysis

**DOI:** 10.3389/fcvm.2024.1375040

**Published:** 2024-07-08

**Authors:** Wenshu Li, Lichaoyue Sun, Sichao Yan

**Affiliations:** ^1^ Department of Pharmacy, Beijing Shijitan Hospital, Capital Medical University, Beijing, China; ^2^ Department of Pharmacy, Aerospace Central Hospital, Beijing, China

**Keywords:** PCSK9 inhibitor, alirocumab, bococizumab, inclisiran, evolocumab, muscle symptom events

## Abstract

**Background:**

Atherosclerotic cardiovascular disease (ASCVD), a leading cause of global fatalities, has inconsistent findings regarding the impact of muscle symptoms despite promising clinical trials involving PCSK9 inhibitors (PCSK9i) and siRNA as potential therapeutic options.

**Methods:**

The databases EMBASE, PubMed, Web of Science, Cochrane, and ClinicalTrials.gov were thoroughly searched without any restrictions on language. Review Manager 5.3 software was utilized to calculate relative risks with 95% confidence intervals (CIs) for dichotomous data and mean differences or standardized mean differences with 95%CIs for continuous data. To evaluate publication bias, Egger's test was employed using Stata/SE software.

**Results:**

This analysis included 26 studies comprising 28 randomized controlled trials (RCTs) involving a total of 100,193 patients, and 4 different lipid-lowering therapy combinations. For events with creatine kinase >3ULN, evolocumab and alirocumab demonstrated significant advantages compared to inclisiran. Evolocumab showed the best results in terms of both new muscle symptom events and creatine kinase >3ULN.

**Conclusions:**

Based on this network meta-analysis (NMA) results, evolocumab has emerged as a promising treatment option for patients with hyperlipidemia and muscle disorders compared to other PCSK9 inhibitors and inclisiran.

**Systematic Review Registration:**

PROSPERO [CRD42023459558].

## 1 Introduction

ASCVD is one of the leading causes of mortality worldwide, accounting for over one-third of all global deaths (1). Dyslipidemia, characterized by the excessive accumulation of low-density lipoprotein cholesterol (LDL-C) in the vasculature, is recognized as a pivotal risk factor in developing ASCVD (2). Consequently, reducing LDL-C levels is essential for managing ASCVD. Statin therapy had been suggested as the first-line therapy by the American College of Cardiology/American Heart Association (ACC/AHA) guidelines and the European Atherosclerosis Society/European Society of Cardiology (EAS/ESC) guidelines. Despite the widespread use of statin therapy, some patients are unable to tolerate it from the outset (3, 4).A meta-analysis identified the most common reason for statin discontinuation as the development of muscle symptoms, with or without changes in creatine kinase (CK) levels. These symptoms occurred in patients who were on increasing doses of statin therapy or who were using a combination of more than two statins (5). As a result, some new therapies have been developed to enhance LDL-C reduction in high-risk ASCVD patients, including PCSK9i and siRNA therapies (6). Achieving guideline-recommended LDL-C goals in statin-intolerant patients requires the use of personalized lipid-lowering therapies other than statins (7). According to the 2018 AHA/ACC guideline and the 2017 National Lipid Association update, PCSK9 inhibitors were recommended for patients with LDL-C levels ≥70 mg/dl or non-high-density lipoprotein cholesterol (non-HDL-C) ≥100 mg/dl after maximally tolerated LDL-lowering therapies (8, 9). The current incidence of statin intolerance is approximately 9.1% and is associated with an increased statin dosage (5).

PCSK9 inhibitors, such as evolocumab, bococizumab, and alirocumab, have demonstrated the ability to bind with PCSK9, effectively inhibiting its interaction with the low-density lipoprotein receptor (LDLR) (10). Common adverse effects of PCSK9 inhibitors include nasopharyngeal pain, headache, and muscle symptoms. Few reports are available comparing the incidence of muscle-related adverse events induced by different types of PCSK9 inhibitors (11). Inclisiran is a siRNA molecule specifically designed to target the mRNA encoding PCSK9, leading to its degradation and the subsequent suppression of PCSK9 protein production (12). The siRNA-mediated degradation of PCSK9 mRNA effectively blocks the synthesis of PCSK9 protein, offering a new therapeutic approach for treating cardiovascular diseases (13, 14). Inclisiran has shown a substantial effect in lowering LDL-C; however, due to the lack of extensive clinical data, its long-term tolerability and safety remain uncertain compared to PCSK9 inhibitors (15). For inclisiran, adverse events at the injection site have been commonly reported. However, the occurrence of muscle symptoms and the elevation of creatine kinase levels have not been thoroughly investigated (16).

For statin-intolerant patients experiencing rhabdomyolysis and requiring alternative therapies, PCSK9i and inclisiran present viable options (17). However, there is no evidence to suggest that these therapies are superior in terms of muscule-related effects. Therefore, we conducted a systematic review and NMA of RCTs to compare the muscle-related adverse effects of these treatments.

## 2 Methods

**Figure 1:**
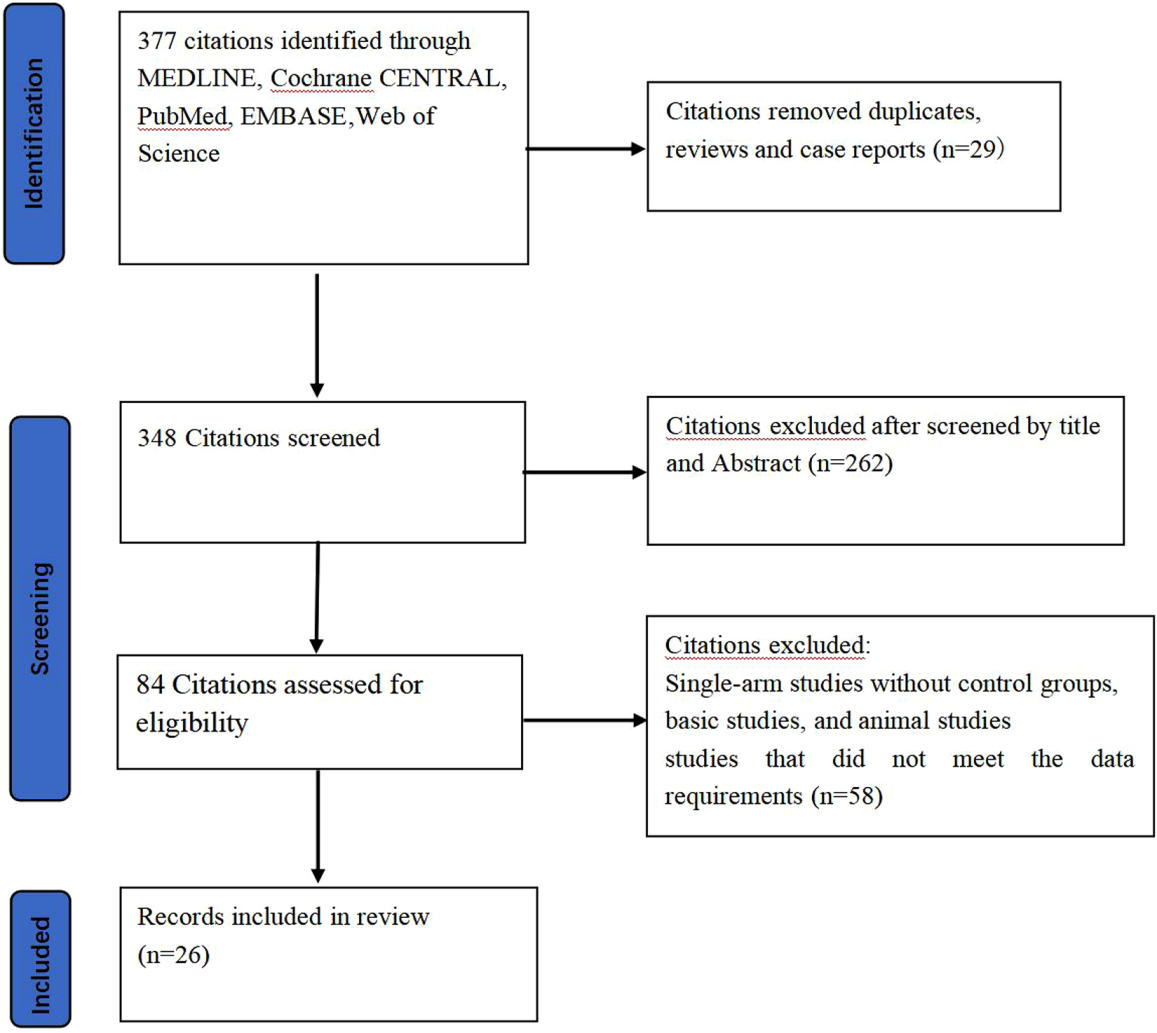
Flowchart. This accompanying flow diagram illustrates the systematic process employed to identify and include pertinent literature in this study.

This NMA followed the guidelines set by the Cochrane Collaboration and was reported by PRISMA (Preferred Reporting Items for Systematic Reviews and Meta-Analysis), as outlined in Figure 1 (18). To ensure the originality, dependability and transparency of the research, the research proposal was registered with the Systematic Review Registry (PROSPERO) under the number CRD42023459558.

### 2.1 Data sources and searches

A detailed literature search was conducted with a language restriction to English using electronic databases including Web of Science, EMBASE, PubMed, Cochrane Library, and Clinical Trials from their inception until December 5, 2023. The search utilized the following keywords: “muscle symptoms,” “creatine kinase,” “inclisiran,” “PSCK9i,” “proprotein convertase subtilisin/kexin type 9 monoclonal antibody,” “PCSK9 inhibitor,” “PCSK9 antibody,” “evolocumab,” “bococizumab,” “alirocumab,” “RG7652,” “AMG145,” “REGN727,” “RN316,” “SAR236553”.

### 2.2 Selection criteria

The studies included in this meta-analysis must adhere strictly to the following criteria:

(1) Eligible studies are Phase II or Phase III RCTs.
(2) The RCTs involved treatment with PSCK9i or inclisiran.
(3) The RCTs report outcomes of new muscular symptoms or CK>3ULN.

The following types of studies were excluded:

(1) Multiple publications describing the same cohort.
(2) Specific categories of publications, including editorial articles, conference abstracts, correspondence, literature reviews, and case reports.
(3) Long-term studies on the safety and effectiveness of PCSK9i replicated in patient cohorts.

### 2.3 Data extraction and quality assessment

All selected trials were processed by the PRISMA guidelines for data extraction. To ensure the highest level of data accuracy and comprehensiveness, three researchers independently extracted the relevant data points. In case of any inconsistencies or uncertainties, discussions were promptly held with a fourth author to reach a consensus, ensuring the accuracy and completeness of the collected data. To maintain the originality and uniqueness of the extracted data, we conducted a thorough review and cross-checked the following information: trial name, sample size, publication year, publication source, first author, trial phase, national clinical trial identification number, number of patients, and intervening measure. In addition to the primary clinical outcomes, we specifically collected and analyzed indicators and incidence rates related to adverse muscular reactions. To ensure the high quality of the included studies we used the Cochrane Risk of Bias tool (1.0) to assess the RCTs (19).

### 2.4 Statistical analysis

To assess the potential impact of PCSK9i therapy on incident muscle symptoms, we conducted meta-analyses using both random- and fixed-effect models to calculate the overall relative risk (RR). Additional details of our data analysis approach were provided in the Supplementary Data. A two-tailed *P* value less than 0.05 was considered statistically significant for the summary treatment effect estimate. All statistical analyses were performed using Stata 16 and Revman (20).

### 2.5 Heterogeneity analysis

To conduct a thorough heterogeneity analysis, we used STATA to calculate the *I*^2^ values, which provide valuable insights into the degree of heterogeneity in this data. An I^2^ value less than 25% indicates low heterogeneity, while values between 25% and 50% denotes moderate heterogeneity. An *I*^2^ value greater than 75% suggests high heterogeneity. In cases of low heterogeneity, we utilized a fixed-effects model to ensure stability and reliability in the analysis. Conversely, when heterogeneity was moderate or high, a random-effects model was employed to account for the broader range of study variations.

We employed the node-splitting method to further assess the consistency of evidence from both direct and indirect sources, ensuring rigorous examines of the internal validity of the evidence synthesis. Additionally, we utilized funnel plots along with Egger's regression test to detect small-study effects, enhancing the comprehensiveness of our evaluation by including a wide range of studies. This approach blosters the reliability and robustness of our findings (21).

## 3 Results

### 3.1 Included studies in the NMA

After an extensive search across four databases (Web of Science, PubMed, Cochrane Library, Embase), we identified 377 relevant articles. After removing duplicates and screening the titles and abstracts, we considered 84 full-text articles for eligibility. The detailed selection process was summarized in Figure 1, including 26 articles in this NMA.

This meta-analysis included 100,193 patients across 28 RCTs, evaluating four lipid-lowing therapies: bococizumab (Boc) (22), evolocumab (Evo) (23–38), alirocumab (Ali) (30, 39–46), and inclisiran (Inc) (47). Basic information for each study, including the first author, publication date, lipid-lowering treatment type, patient sex ratio, age, follow-up duration, NCT number, and patient profile is provided in Supplementary Table S1.

### 3.2 Characteristics of the research reports

**Figure 2:**
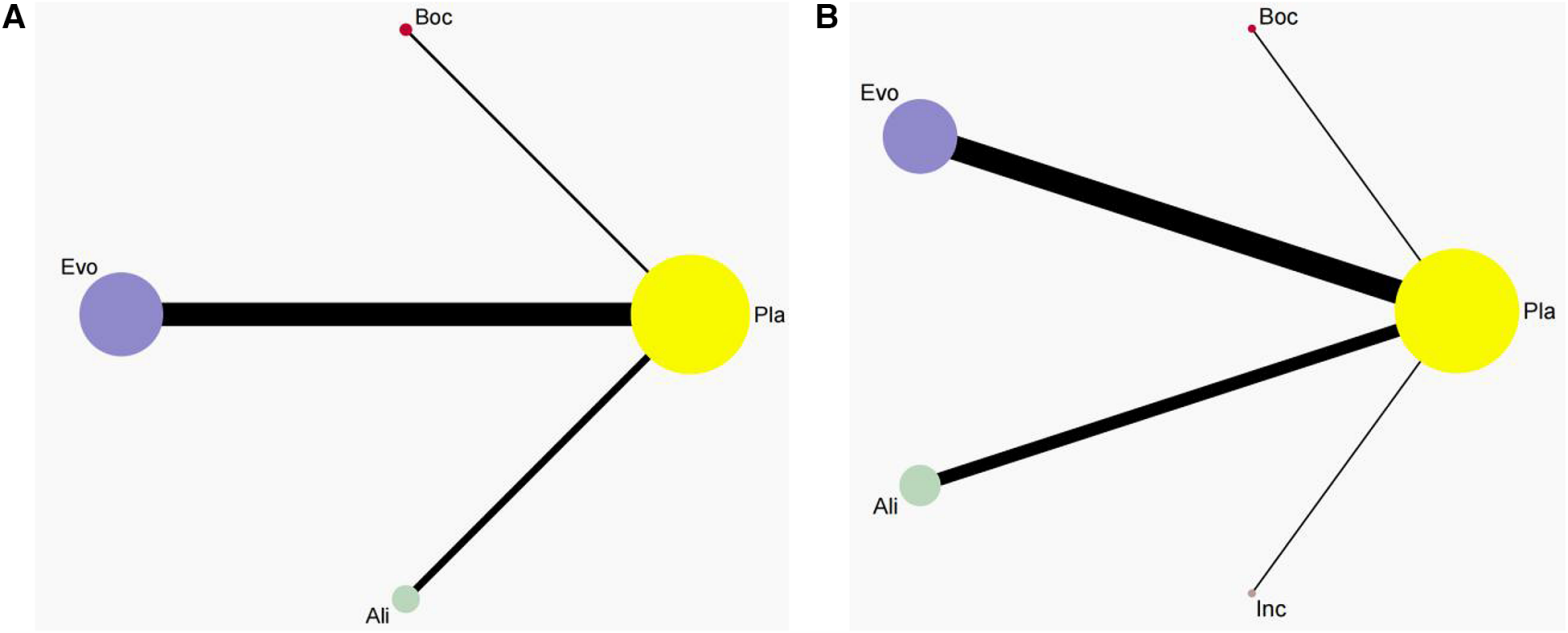
Network plot. This network plot illustrates the safety of three (**A**) and four(**B**) different lipid-lowering therapies (PCSK9i and Inclisiran) for patients. In the plot, circles are used to represent each intervention as a node in the network, while lines depict direct comparisons within the framework of RCTs. The thickness of the lines corresponds to the number of RCTs included in each comparison.

In this analysis, these 26 studies compared bococizumab with placebo (1 study), evolocumab with placebo (16 studies), alirocumab with placebo (9 studies), and inclisiran with placebo (1 study). Additionally, one study examined the safety profiles of both evolocumab and alirocumab compared to placebo controls. Figure 2 provides a visual representation of the eligible comparisons in the form of a network plot.

### 3.3 Assessment of included RCTs

**Figure 3:**
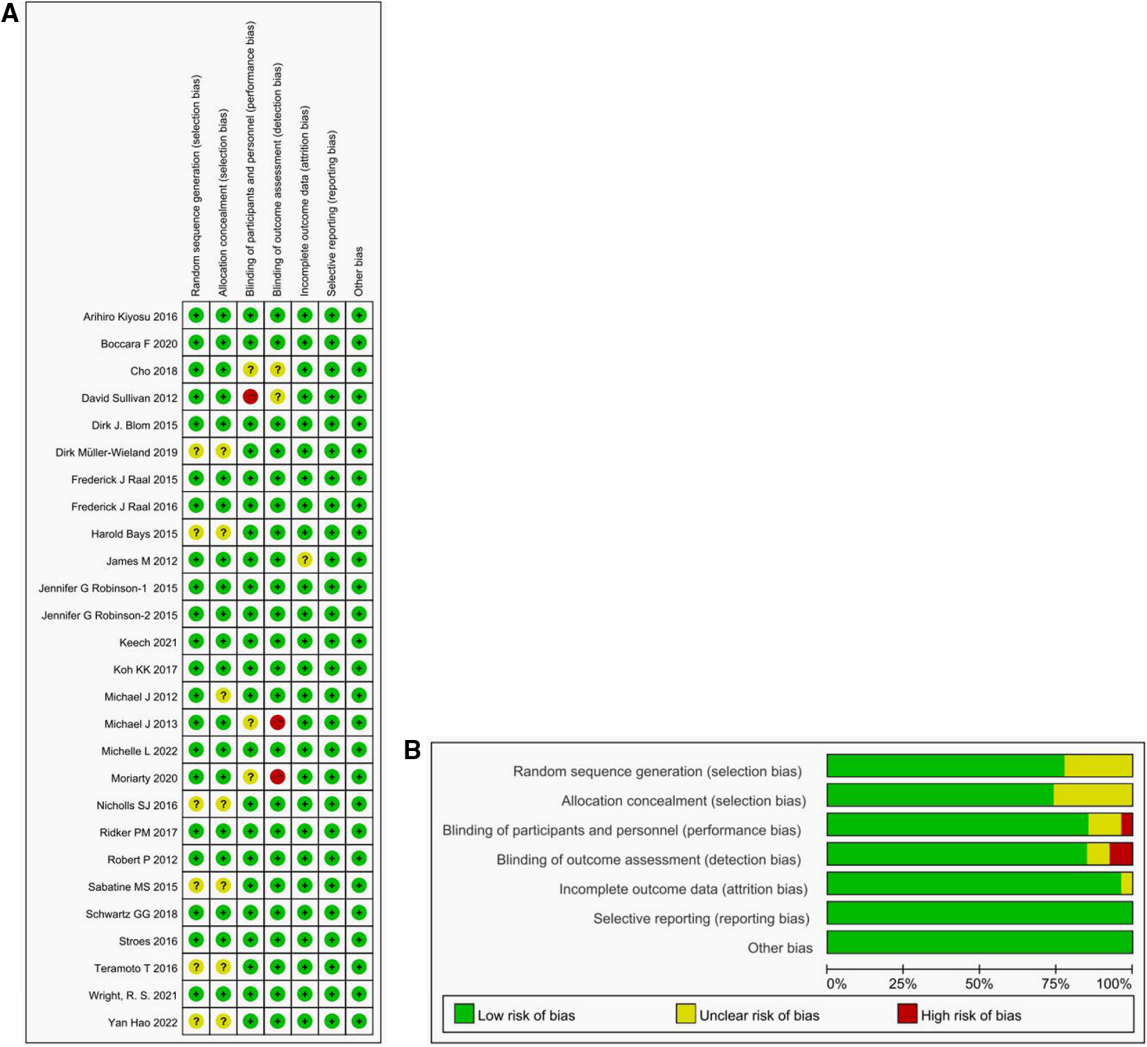
Risk of bias figure. (**A**) Methodological Quality Summary: This section presents the authors’ assessment of each methodological quality item for each study included in the analysis. The two main sources of bias evaluated are performance bias and detection bias. (**B**) Methodological Quality Map: We provide their evaluation of the overall quality of each methodology used in the included studies, expressed as a percentage of all included studies. Described as a percentage of all included studies.

Figure 3 presents the outcomes of the risks of bias assessment for the 26 trials included in the study. Overall, the risk of bias was considered low due to the robust design of the RCTs employed. To further ensure methodological rigor, we also reviewed the test protocols for additional details.

Regarding random sequence generation, 20 studies were assessed as having a low risk, while 6 studies had an unclear risk. For allocation concealment, 19 studies had a low risk, and 7 studies had an unclear risk. In terms of performance bias, 22 studies examined a low risk, 3 studies had an unclear risk, and 1 study had a high risk. For detection bias, 22 studies had a low risk, 2 studies had an unclear risk, and 2 studies had a high risk. When evaluating attrition bias, 25 studies were considered to have a low risk, while 1 study had an unclear risk. All trials were rated as having a low risk for the reporting bias, primarily because the data analysis focused on the intention-to-treat population and included an adequate number of relevant endpoints. However, it is worth noting that some studies allowed for crossover, which could introduce potential biases into the results.

### 3.4 Pairwise meta-analysis

Pairwise meta-analyses were conducted for 22 trials reporting new muscle symptoms and for 22 trials reporting events of creatine kinase >3ULN.

Head-to-head comparisons revealed that, compared to placebo, patients treated with bococizumab experienced a higher incidence of muscle symptoms. (RR = 1.09; 95%CI: 0.95–1.25, *P* = 0.22) and creatine kinase >3ULN (RR = 0.86; 95%CI: 0.68–1.09, *P* = 0.22). Similarly, evolocumab increased the risk of muscle symptoms (RR = 1.05; 95%CI: 0.97–1.14, *P* = 0.94) and creatine kinase >3ULN (RR = 0.69; 95%CI: 0.43–0.96, *P* = 0.26). Additionally, alirocumab elevated the risk of muscle symptoms (RR = 1.16; 95%CI, 0.89–1.51, *P* = 0.28) and creatine kinase >3ULN (RR = 0.86; 95%CI: 0.66–1.12, *P* = 0.27). Inclisiran solely heightened the risk of creatine kinase >3ULN (RR = 1.09; 95%CI:0.61–1.93, *P* = 0.78).

**Figure 4:**
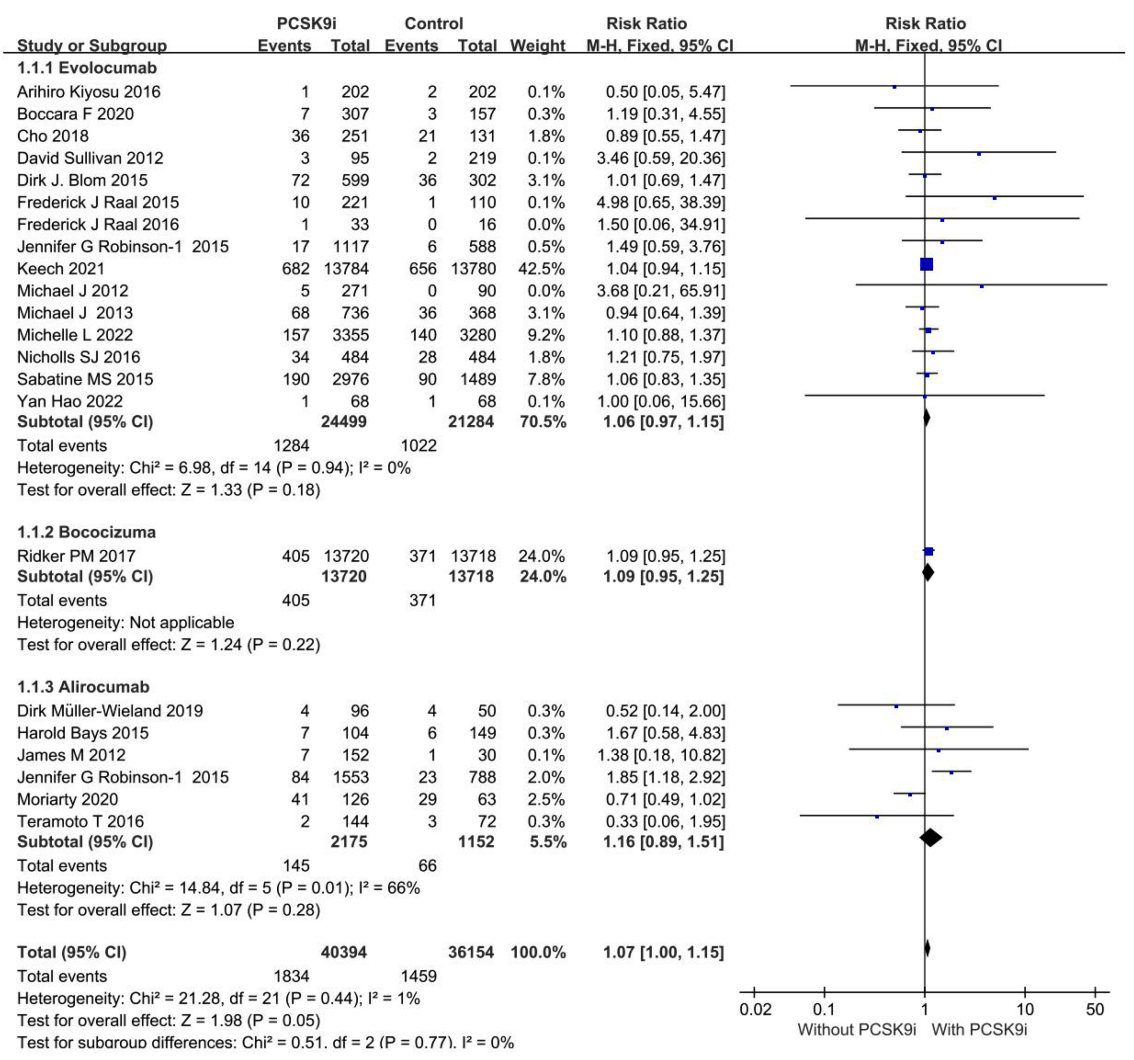
Forest plot. Forest plot for new muscle symptom events. The safety of PCSK9i in hyperlipidemic patients.

**Figure 5:**
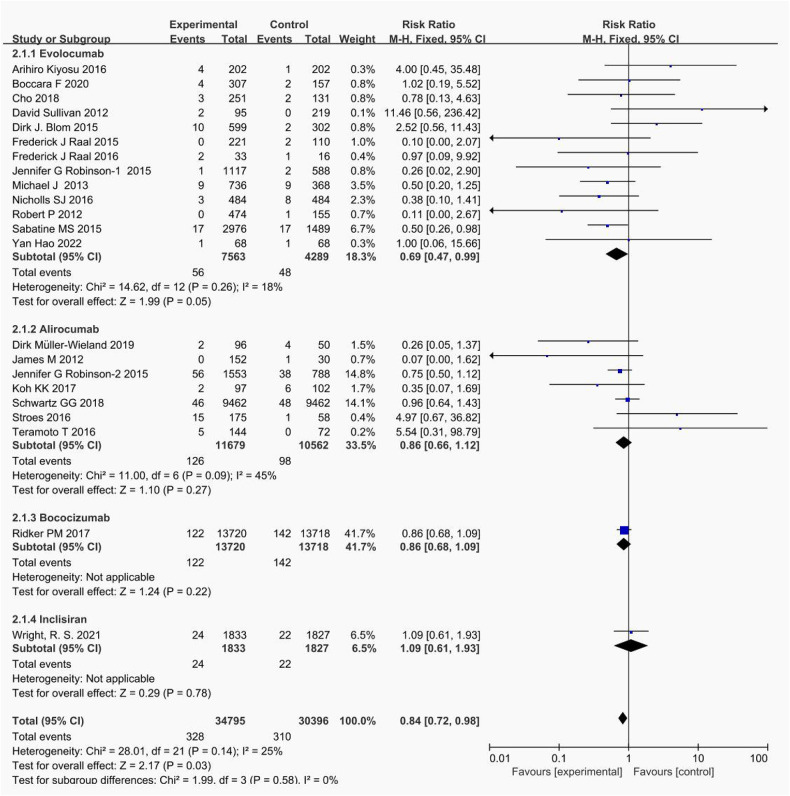
Forest plot. Forest plot for events with creatine kinase >3ULN.The safety of PCSK9i and inclisiran in hyperlipidemic patients.

The forest plots in Figures 4, 5 visualize the pairwise comparisons of the incidence of muscle symptom and creatine kinase >3ULN, respectively. As shown in Supplementary Figures S1, S2, the funnel plot shows no significant publication bias in this study.

### 3.5 Network meta-analysis

**Figure 6:**
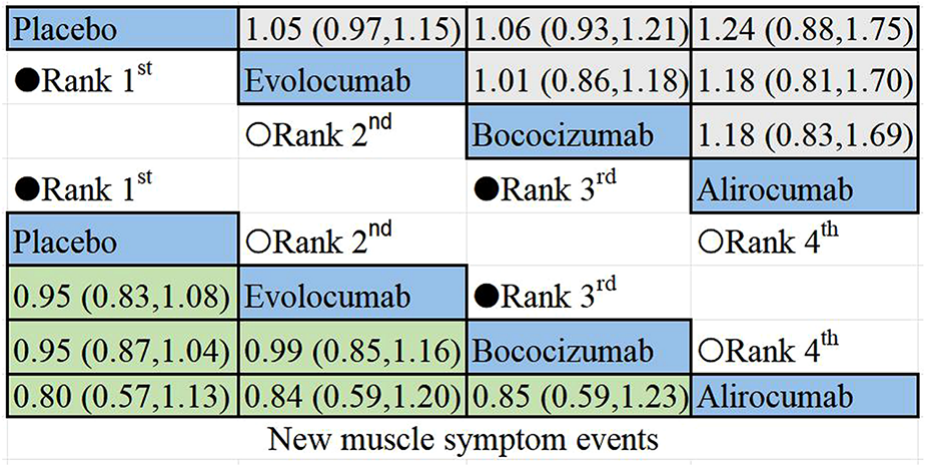
Summary of target outcomes including new muscle symptom events. Safety of PCSK9i in hyperlipidemic patients analyzed by Bayesian network meta-analysis.

The non-direct comparative results for new muscle symptom events are displayed in Figure 6. Among PCSK9i, alirocumab posed the highest risk for new onset muscle symptoms, followed by bococizumab, with evolocumab being the least likely to cause such events.

**Figure 7:**
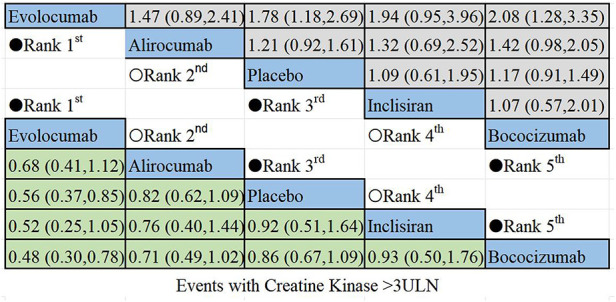
Summary of target outcomes including events with creatine kinase >3ULN. Safety of PCSK9i and inclisiran in hyperlipidemic patients analyzed by Bayesian network meta-analysis.

The results of the non-direct comparisons for events with creatine kinase >3ULN are presented in Figure 7. Compared to inclisiran, bococizumab (RR = 1.07; 95%CI: 0.57–2.01), evolocumab (RR = 0.52;95% CI: 0.25–1.05), alirocumab (RR = 0.76; 95%CI: 0.4–1.44), and placebo (RR = 0.92; 95% CI: 0.51–1.64) exhibited varying risk patterns. The order of lipid-lowering agents causing new-onset CK>3ULN in descending order of risk: bococizumab > inclisiran > placebo > alirocumab > evolocumab. Evolocumab appeared to carry the lowest risk for elevated creatine kinase levels, while bococizumab posed the highest risk among the lipid-lowering agents.

### 3.6 Subgroup meta-analysis

**Figure 8:**
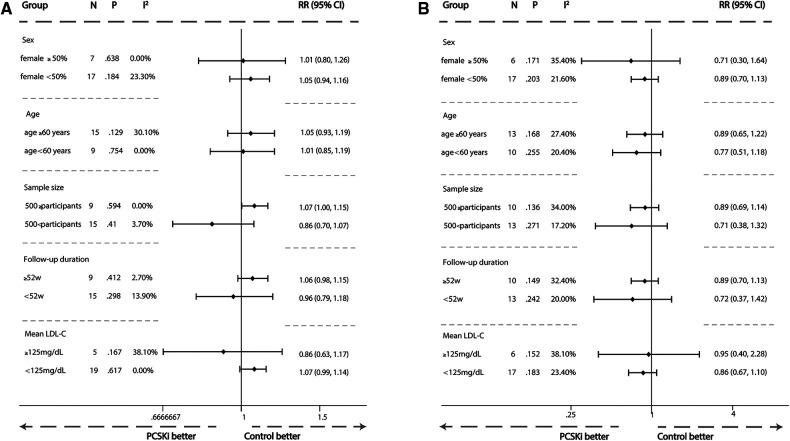
Subgroup meta-analysis of association between PCSK9i therapy and risk of new muscle symptom and creatine kinase >3ULN. (**A**) Subgroup meta-analysis of association between PCSK9i therapy and risk of incident muscle symptoms. (**B**) Subgroup meta-analysis of association between PCSK9i and inclisiran therapy with risk of incident Creatine Kinase >3ULN.

A subgroup analysis was conducted to evaluate the risk of muscle adverse events and creatine kinase elevation caused by PCSK9i and inclisiran from five perspectives: age (≥60 years or <60 years), gender (female ≥50% or female <50%), different LDL-C level before treatment (≥125 mg/dl or <125 mg/dl), follow-up time (≥52 weeks or <52 weeks), and sample size (≥500 participants or <500 participants) in Supplementary Figures S3–S6. The result showed that gender, age, LDL-C level before treatment, follow-up time, and sample size had no significant impact on the risk of muscle adverse events and creatine kinase elevation caused by PCSK9i and inclisiran in Figure 8.

## 4 Discussion

In this comprehensive NMA, encompassing a substantial cohort of 100,193 patients who either received high-dose statin treatment or reported intolerance to statins, our results indicated that the use of inclisiran and PCSK9i may lead to various adverse effects throughout therapy. Previous reports had suggested that these lipid-lowering therapies could impact the neurocognitive system of patients or even increase the risk of fractures (48, 49). However, the risk of muscular adverse events associated with PCSK9i and inclisiran had not been comprehensively summarized in a complete meta-analysis until now.

The utilization of NMA represented an advancement compared to traditional meta-analyses, as it allows for indirect comparisons of interventions across RCTs by incorporating a joint comparator group. This approach encompassed a broader range of studies, thereby enhancing the credibility of the findings. When evaluating the relative safety profiles of PCSK9i (such as evolocumab, bococizumab, and alirocumab) and inclisiran in patients with hyperlipidemia, head-to-head clinical trials are invaluable. They provide essential insights that guide clinical decision-making.

The comparison of new muscular symptom events demonstrated that evolocumab exhibited the highest level of safety, followed by bococizumab. In contrast, patients treated with alirocumab showed a relatively higher incidence of new muscular symptoms.

Similarly, when comparing events with CK>3ULN, patients treated with bococizumab had a higher risk of elevated creatine kinase compared to those treated with inclisiran (22, 47). In contrast, other PCSK9 inhibitors, such as evolocumab and alirocumab, demonstrated a better safety profile than inclisiran. with evolocumab having the fewest incidents of creatine kinase elevation. However, it is important to note that inclisiran and bococizumab were each included in only one trial, which may impact the results of the NMA. More RCTs are needed in the future to confirm these findings. Notably, bococizumab has been suspended in recent years due to its higher immunogenicity (50).

Adverse drug reactions were observed to be both more severe and more frequent in female subjects compared to their male counterparts. The pharmacological aspects of these reactions have been comparatively understudied. To develop appropriate individualized dosing regimens, gender differences should receive greater attention (51). This may require additional clinical trials to validate this observation. Moreover, an interesting phenomenon was noted, patients with specific genotypes (e.g., SLCO1B1rs4149056) had more difficulty reaching the LDL-C target value, with a notable gender difference in this effect. Future studies should focus on the safety and efficacy of PCSK9 inhibitors in genetically diserve patients to further explore these differences (52).

Through this comprehensive NMA, we provided valuable insights into the relative safety of PCSK9i and inclisiran in patients with hyperlipidemia. These findings have important implications for clinical decision-making and patient outcomes. It is significant to note that clinicians might face challenges in selecting these therapies due to the high cost of PCSK9 inhibitors. A cost-effectiveness analysis of PCSK9 inhibitors and inclisiran would provide a crucial basis to supporting their use in statin-intolerant patients (53).

## 5 Strengths and limitations

Our analysis offered significant insights into the safety of muscle adverse events among patients using different lipid-lowering therapies. However, it is imperative to recognize the necessity for additional studies to validate and expand upon our findings. Considering the intricate and diverse nature of individuals with hyperlipidemia, it is crucial to personalize treatment decisions on an individual basis. Patient stratification based on factors such as ethnicity and age may play a vital role in selecting the most suitable lipid-lowering drug for each patient. By tailoring treatment protocols to align with the distinct clinical profiles of individual patients, we can enhance therapeutic outcomes and reduce the incidence of novel muscule symptoms.

Notably, the limitations of these findings stem from the quality of available evidence, including internal bias and heterogeneity. Incomplete reporting, controversial treatment classifications, and potential misclassification also posed constraints. Furthermore, high-impact interventions might be influenced by other factors associated with higher socioeconomic status in the patient population. Therefore, further exploration of these potential limitations is crucial for enhancing our understanding of the safety lipid-lowering therapy in patients with hyperlipidemia.

## 6 Conclusions

Based on the results of this NMA, evolocumab demonstrated the lowest likelihood of causing adverse muscle effects compared to other PCSK9 inhibitors (bococizumab, alirocumab) and inclisiran.This makes evolocumab a promising lipid-lowering option for patients with both hyperlipidemia and muscle disease.

## Data Availability

The original contributions presented in the study are included in the article/**Supplementary Material**, further inquiries can be directed to the corresponding author.
