# Supplementary material for: PCSK9 inhibitors and inclisiran with or without statin therapy on incident muscle symptoms and creatine kinase: a systematic review and network meta-analysis
Source: Front Cardiovasc Med. 2024 Jul 8;11:1375040. doi: 10.3389/fcvm.2024.1375040 (PMC11260805; doi:10.3389/fcvm.2024.1375040)
Supplement: Supplementary file 1 [file Datasheet1.docx]

Supplementary Material

# Supplementary Figures and Tables

## Supplementary Figures

|  | | | | | | | | | |
| --- | --- | --- | --- | --- | --- | --- | --- | --- | --- |
| Ref. | RCT Phase | Drug | Dose | N | Male(%) | Age  (years) | Follow-up  length | Patient profile | NCT number |
| David Sullivan, et al,2012 | II | Evolocumab  Ezetimibe | 280 mg, 350 mg, or 420 mg q4w  10mg qd | 95  62 | 37.9  33.9 | 61.5  62.2 | 12w | Hypercholesterolemia with statin intolerance | NCT01375764 |
| Michael J Koren, et al,2012 | II | Evolocumab  Ezetimibe  Placebo | 70mg /up to 150 mg q2w ,  280mg/up to 420mg q4w  10mg qd | 271  45  90 | 31.4  42  38.9 | 50.4  50.0  51.6 | 12w | Serum LDL-C concentrations of 2·6 mmol/L or greater but less than 4·9 mmol/L | NCT01375777 |
| Robert P Giugliano,et al,2012 | II | Evolocumab  Placebo | 70mg /up to 150 mg q2w  280mg/up to 420mg q4w | 474  157 | 62.1  62.0 | 49.1  46.5 | 12w | Hypercholesterolaemia and fasting LDL-C concentration greater than 2·2 mmol/L while on a stable dose of statin (with or without Ezetimibe) for at least four weeks | NCT01380730. |
| James M. McKenney, et al.2012 | II | Alirocumab  Placebo | 50mg /up to 150 mg q2w  200mg or 300mg q4w | 152  31 | 46.7  51.6 | 57.4  53.3 | 20w | Men and non-pregnant, nonlactating women aged 18 to 75 years (incl sive), with LDL-C 100 mg/dl (2.59 mmol/l) while receiving a stable dose of atorvastatin 10, 20, or 40 mg daily for 6 weeks. | NCT01288443 |
| Leslie Cho,et al.2013 | III | Evolocumab  Ezetmibe | 140mg q2w or 420mg q4w  10mg qd | 205  102 | 55.1  52.0 | 62.0  61 | 14w | Hypercholesterolemia with statin intolerance | NCT01763905 |
| Michael J. Koren ，et al.2013 | III | Evolocumab SOC | 420mg q4w | 736  368 | N/A | N/A | 52w | Patients completing any evolocumab phase 2 parent study | NCT01439880 |
| Jennifer G. Robinson,et al.2015 | III | Evolocumab  Ezetimibe  Placebo | 140mg q2w or 420mg q4w  10mg qd | 1117  221  558 | 56  50.7  52.2 | 59.6  60.8  59.9 | 12w | LDL-C level of 150 mg/dL or greater, 100 mg/dL or greater, or 80 mg/dL or greater, fasting triglyceride levels of 400 mg/dL or less, and use an intensive statin. | NCT01763866 |
| Dirk J. Blom, et al. 2015 | III | Evolocumab  Placebo | 420mg q4w | 599  302 | 48.4  46.4 | 55.9  56.7 | 52w | LDL cholesterol level of 75 mg per deciliter (1.94 mmol per liter) or higher and a fasting triglyceride level of 400 mg per deciliter (4.52 mmol per liter) or lower. | NCT01516879 |
| Bays H, et al.2015 | III | Alirocumab  Ezetimibe  placebo | 75/150mg q2w  10mg qd | 104  102  149 | 61.5  65.7  67.1 | 63.1  64.9  61.4 | 24w | High CVD risk and LDL-C of 70 mg/dL or greater or at high risk | [NCT01730040](https://clinicaltrials.gov/ct2/show/NCT01730040) |
| Schwartz GG, et al. 2015 | III | Alirocumab  Placebo | 150mg q2w | 1553  788 | 63.3  60.2 | 60.4  60.6 | 78w | Heterozygous for FH or established CHD or CHD risk equivalent | NCT01507831 |
| Frederick J Raal, et al. 2015 | III | Evolocumab  Placebo | 420mg q4w | 33  16 | 52  50 | 30±12  32±14 | 12w | Homozygous familial hypercholesterolemia | NCT01588496 |
| Frederick J Raal, et al. 2015 | III | Evolocumab  Placebo | 140mg q2w or 420mg q4w | 220  109 | 55.0  59.1 | 52.3  48.9 | 12w | Homozygous familial hypercholesterolemia | NCT01763918 |
| Sabatine MS, et al. 2015 | III | Evolocumab  Standard therapy | 140 mg q2w or 420 mg q4w | 2976  1489 | 57.8  58.2 | 50.1  51.4 | 52w | Patients who had completed one of the parent studies could enroll in one of the OSLER extension studies. | NCT01439880  NCT01854918 |
| Teramoto T, et al.2016 | III | Alirocumab  Placebo | 75 /up to 150 mg q2w | 144  72 | 58.3  65.3 | 60.3  61.8 | 52w | Heterozygous for FH, non-FH at high CV risk with coronary disease or JAS category III | NCT02107898 |
| Arihiro Kiyosu, et al. 2016 | III | Evolocumab  Placebo | 140mg q2w or 420mg q4w | 202  202 | 60  61 | 62±11  61±10 | 12w | Eligible patients (aged ! 20 and 85 years) from study sites in Japan were at high risk for CV events based on Japan Atherosclerosis Society (JAS) criteria | NCT01953328 |
| Nicholls SJ, et al. 2016 | III | Evolocumab  Placebo | 420mg q4w | 484  484 | 72.1  72.3 | 59.8  59.8 | 72w | 1. At least 1 epicardial coronary stenosis of 20% or greater on clinically indicated coronary angiography and had a target vessel suitable for imaging with 50% or less visual obstruction. 2. Treated with a stable statin dose for at least 4 weeks | NCT01813422 |
| Steven E. Nissen, et al. 2016 | III | Evolocumab  Ezetmibe | 420mg q4w  10mg qd | 145  73 | 53.8  46.6 | 59  58.5 | 24w | Hypercholesterolemia with muscle-related statin intolerance | NCT01984424 |
| Erik Stroes, et al. 2016 | III | Alirocumab | 75 mg q2w/up 150 mg q2w  300 mg q4w/up 150 mg q2w | 116  59 | 59.5  50.8 | 62.5  64.2 | 24w | Very high or high CV risk with inadequately controlled hypercoaster-olema | NCT02023879 |
| Ridker PM，et al. 2017 | III | Bococizumab  Placebo | 150 mg q2w | 13716  13718 | 70.7  70.2 | 63.3  62.4 | 144w | A history of cardiovascular disease or a high risk of cardiovascular disease | NCT01975376  NCT01975389 |
| Koh KK et al. 2017 | III | Alirocumab  Placebo | 75 /up to 150 mg q2w | 97  102 | 85.6  79.4 | 61.2  60.1 | 24w | Hypercholesterolemia at high CV risk and on maximally tolerated statin | NCT02289963 |
| Leslie Cho, et al.2018 | III | Evolocumab  SOC | 140mg q2w or 420mg q4w | 251  131 | 49.8  50.4 | 60.9 ±9.1 61.9 ±9.2 | 52w | Patients who completed GAUSS-1 and GAUSS-2 | NCT01439880 |
| Schwartz GG, et al. 2018 | III | Alirocumab  Placebo | 75 /up to 150 mg q2w | 9462  9462 | 74.7  74.9 | 58.5  58.6 | 256w | Inadequately controlled hypercholesterolemia under treatment of statin and had an acute coronary syndrome 1–12 months earlier | NCT01663402 |
| Dirk Müller-Wieland, et al. 2019 | III | Alirocumab  Placebo | 75 mg q2w/up 150 mg q2w  300 mg q4w/up 150 mg q2w | 115  458 | 37.8/65.4  45.2/60.9 | 59.3/60.7  59.2/61.6 | 48w | Moderate-to-very-high CV risk with inadequately controlled hypercoaster-olema | NCT01926782 |
| Moriarty, et al.2020 | III | Alirocumab  placebo | 75 mg q2w/up 150 mg q2w  300 mg q4w/up 150 mg q2w | 116  59 | 59.5  50.8 | 62.5  64.2 | 24w | Very high or high CV risk with inadequately controlled hypercoaster-olema | NCT01709513 |
| Boccara F, et al. 2020 | III | Evolocumab  Placebo | 420 mg q4w | 307  157 | 85.7  76.4 | 56.4  56.2 | 24w | HIV-infected patients with dyslipidemia taking maximally-tolerated statin therapy | NCT02833844 |
| Keech, A, et al.2021 | III | Evolocumab | 140 mg q2w or 420 mg q4w | 13784 | 75.4 | 62.5 | 104w | Hypercholesterolemia with statin use and ASCVD | NCT02833844 |
| Wright, R. S, et al. 2021 | III | Inclisiran  Placebo | 300mg Day1，Day90，then every 6 months | 1833  1827 | 66.9  68.1 | 64.1±9.98  63.9±9.87 | 72w | Hyperlipidemia with high CV risk and receiving the maximum tolerated statin therapy | NCT03397121  NCT03399370  NCT03400800 |
| Michelle L. O’Donoghue, et al. 2022 | III | Evolocumab  Placebo | 140mg q2w or 420mg q4w | 3355  3280 | 77.0  76.3 | 62.4  62.4 | 12w | Hyperlipidemia | NCT02867813 NCT03080935 |
| Yan Hao, et al.2022 | III | Evolocumab  Ezetimibe | 140mg q2w or 420mg q4w  10mg qd | 68  68 | 66.18  70.59 | 62.21±12.31  62.22±11.44 | 12w | High-risk ACS with high levels of LDL-C | N/A |
| N/A: Not applicable; SOC: standard of care | | | | | | | | | |

**Supplementary Table 1.** Baseline characteristics of the studies included in the network Meta-analysis.


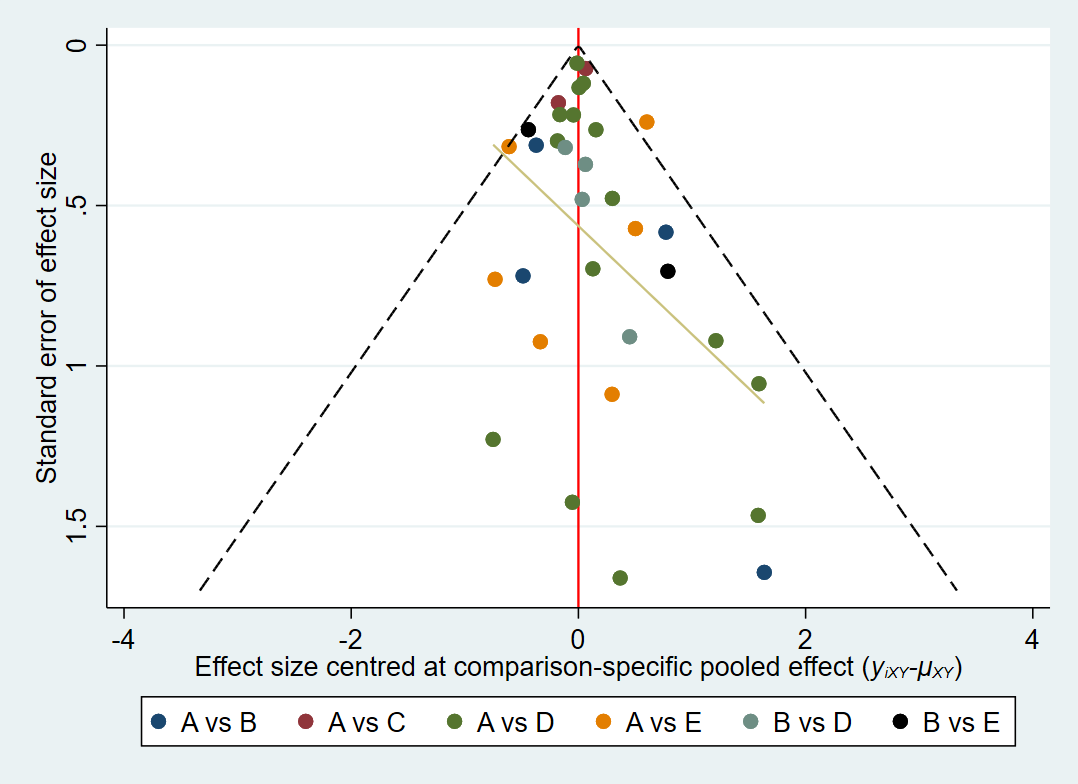


**Supplementary Figure 1. Funnel plots for New Muscle Symptom Events.**


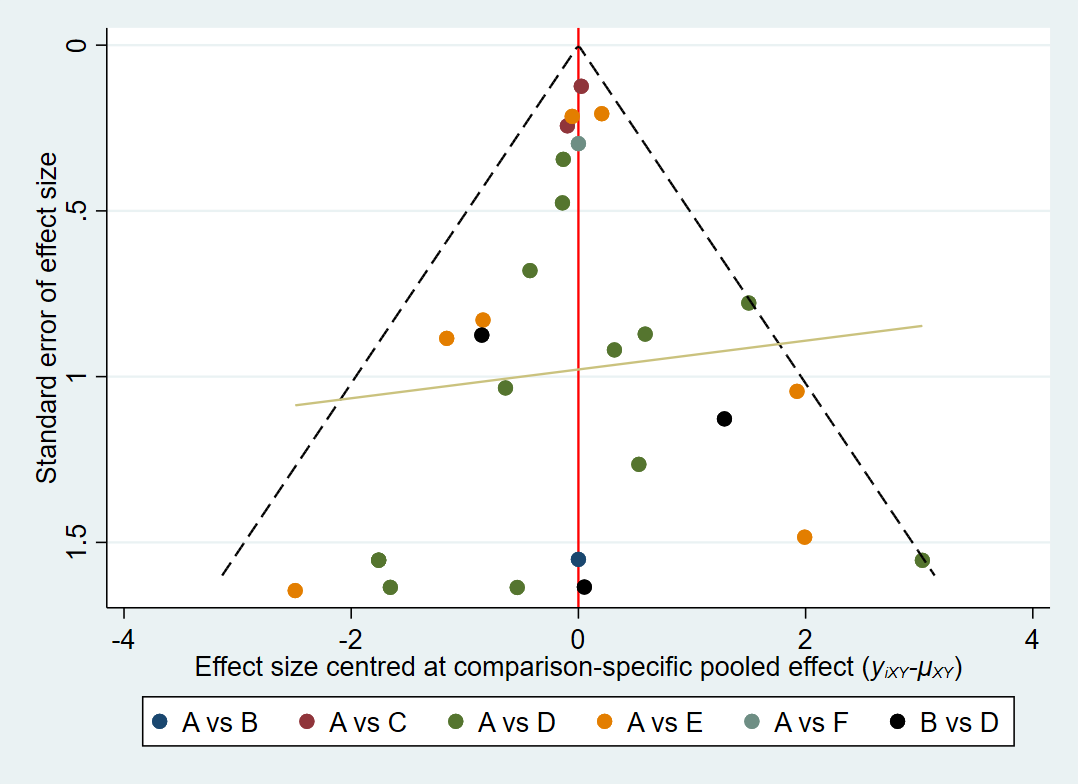


**Supplementary Figure 2. Funnel plots for Events with Creatine Kinase >3ULN.**


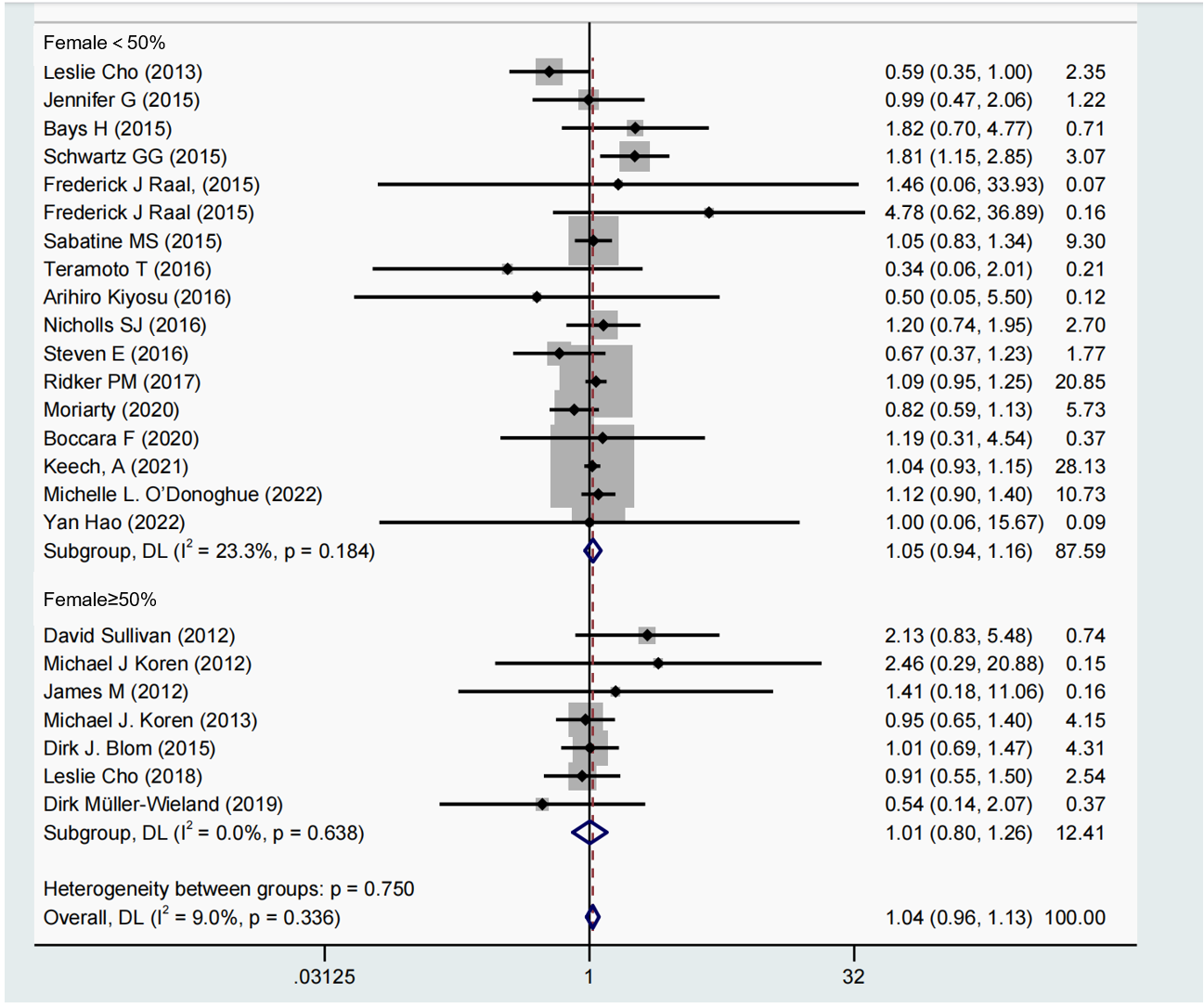


**Supplementary Figure 3A. Forest plot for Sex gender.The risk of New Muscle Symptom of PCSK9i and Ezetimibe in hyperlipidemic patients.**


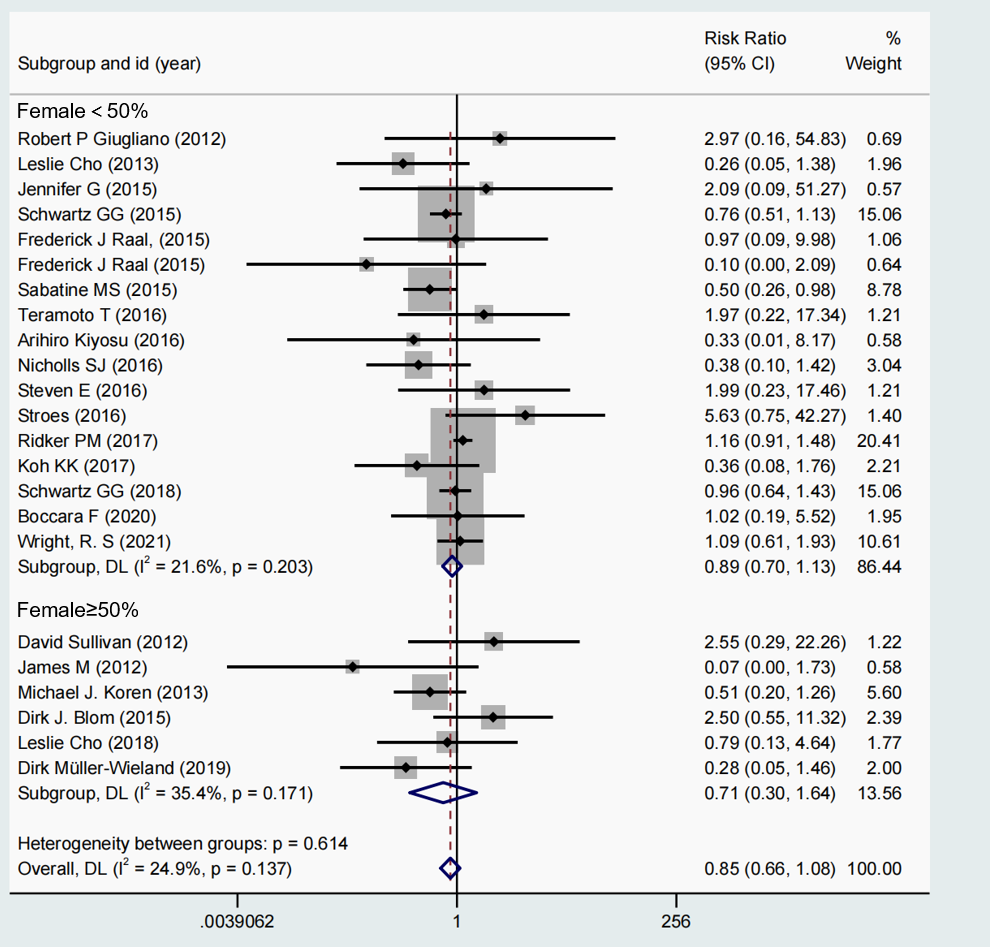


**Supplementary Figure 3B. Forest plot for Sex gender.The risk of Creatine Kinase >3ULN of PCSK9i and Ezetimibe in hyperlipidemic patients.**


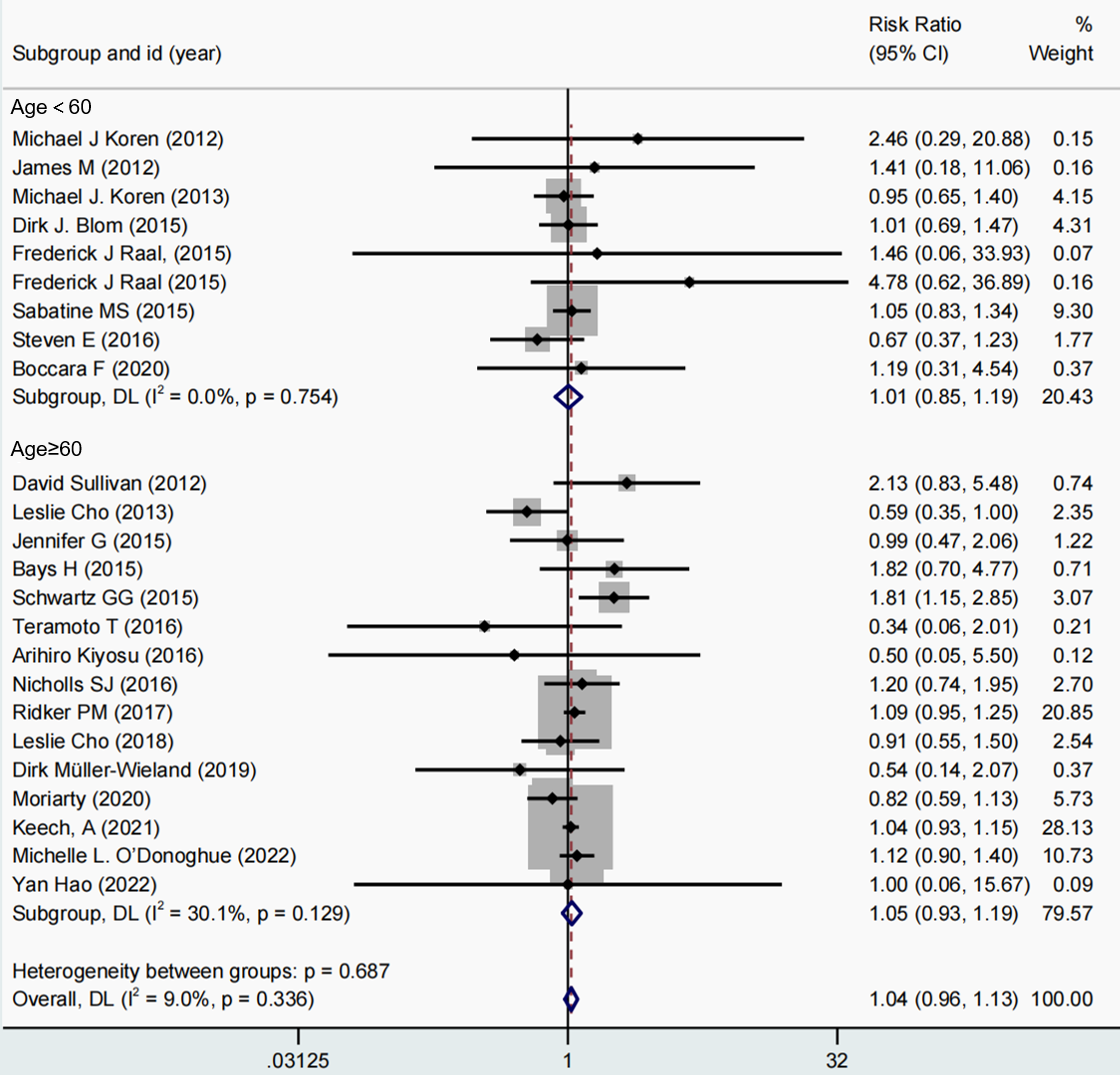


**Supplementary Figure 4A. Forest plot for age.The risk of New Muscle Symptom of PCSK9i and Ezetimibe in hyperlipidemic patients.**


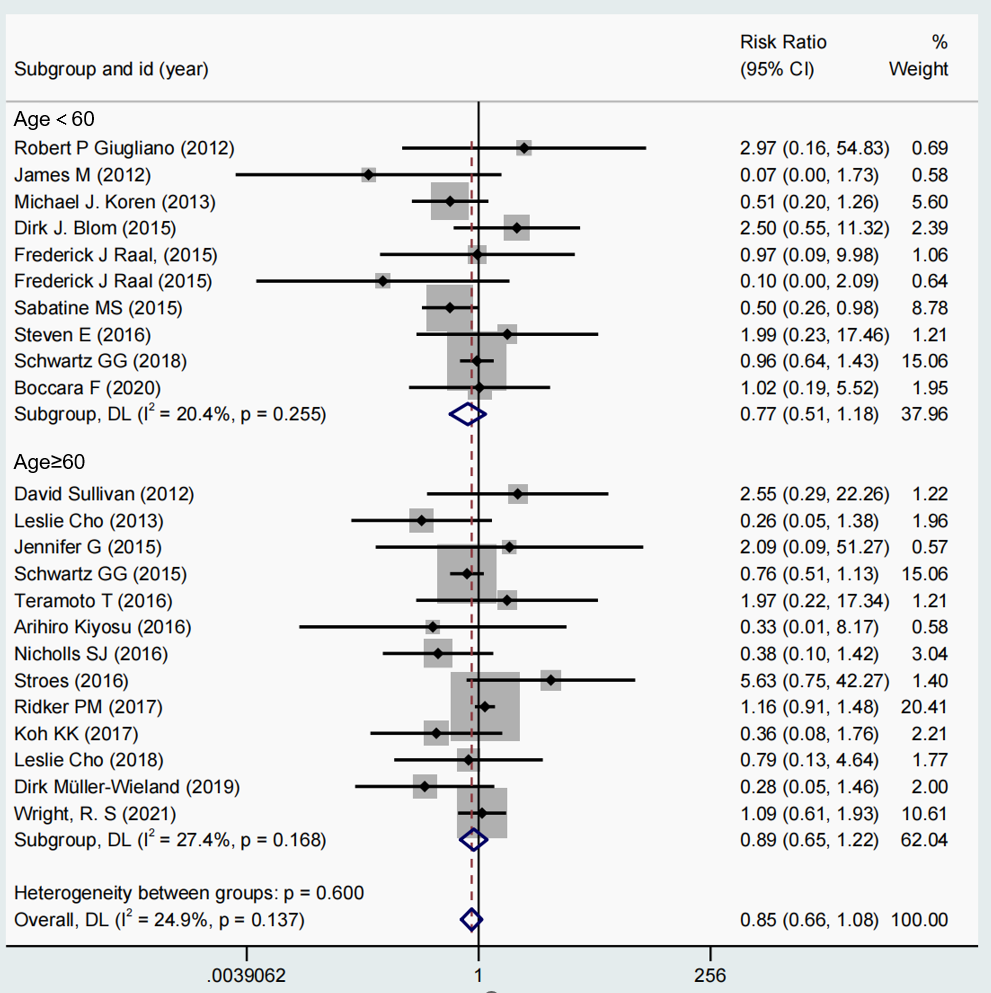


**Supplementary Figure 4B. Forest plot for age.The The risk of Creatine Kinase >3ULN of PCSK9i and Ezetimibe in hyperlipidemic patients.**


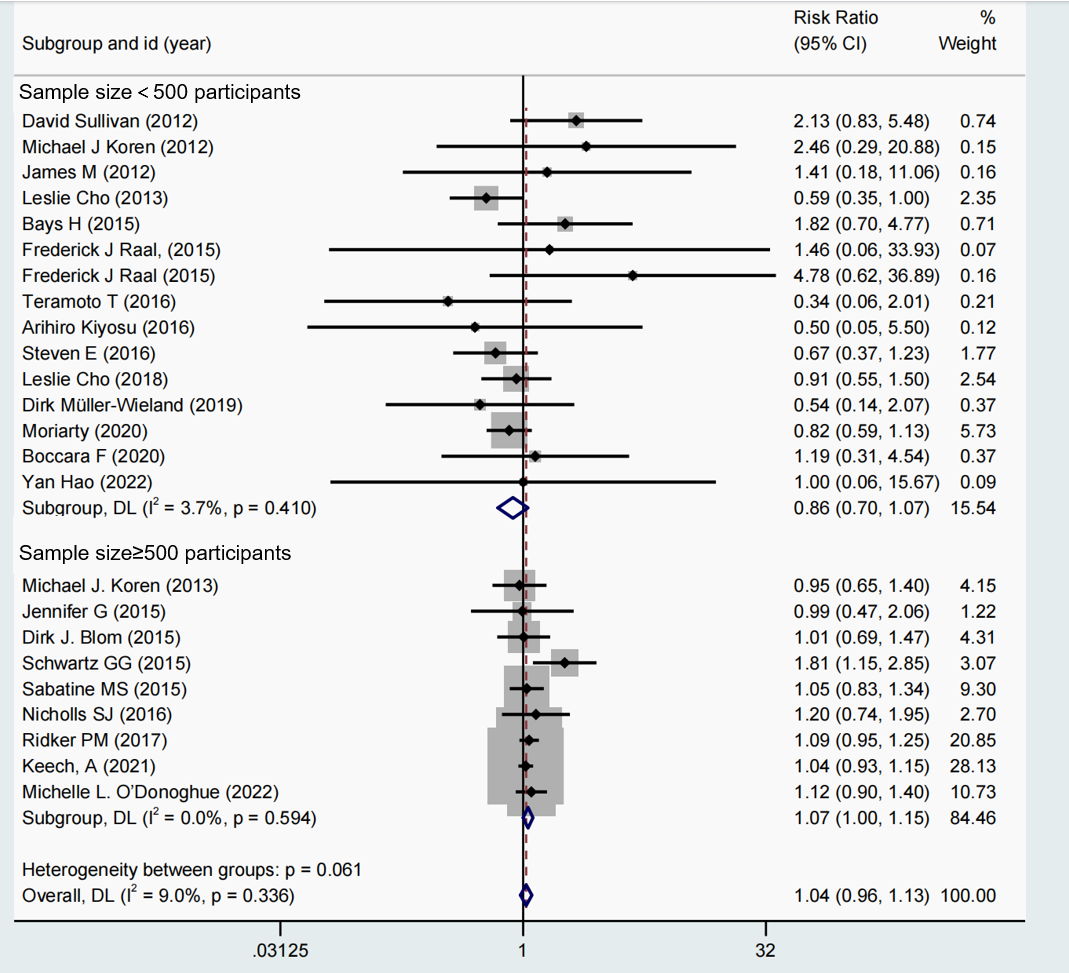


**Supplementary Figure 5A. Forest plot for sample size.The risk of New Muscle Symptom of PCSK9i and Ezetimibe in hyperlipidemic patients.**


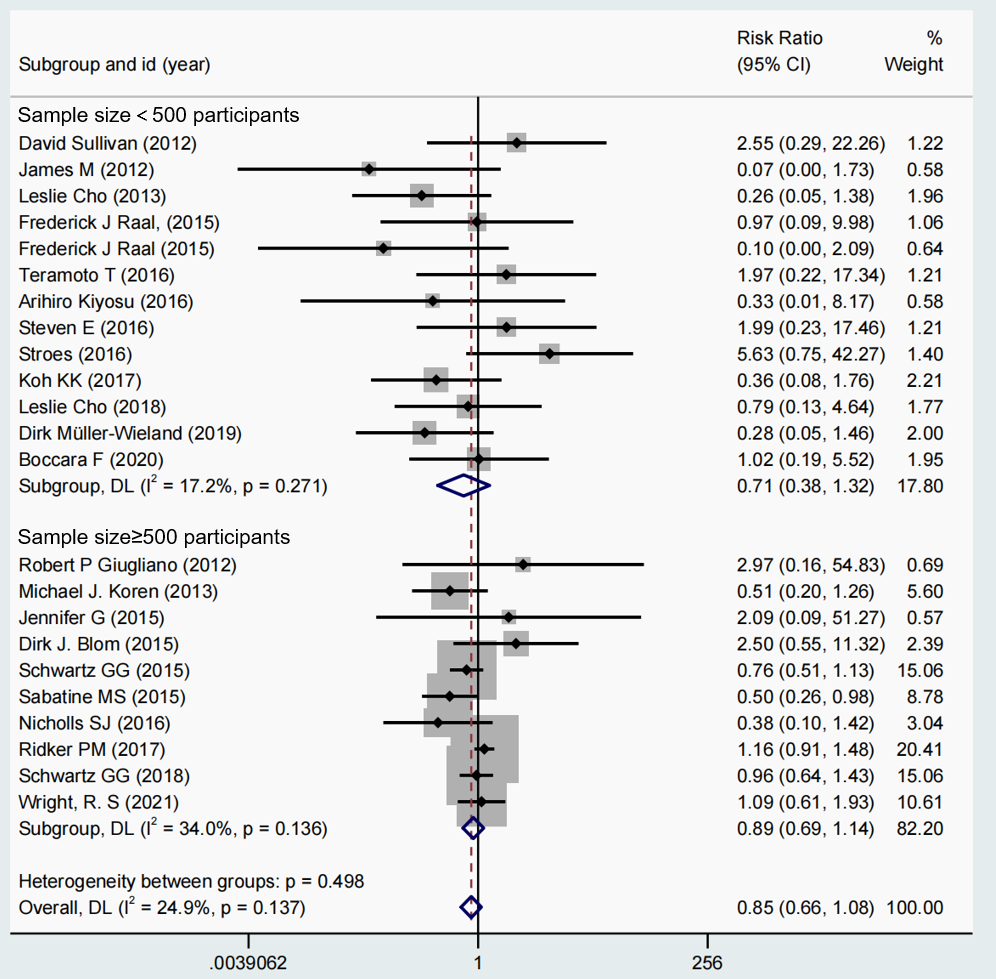


**Supplementary Figure 5B. Forest plot for sample size.The The risk of Creatine Kinase >3ULN of PCSK9i and Ezetimibe in hyperlipidemic patients.**


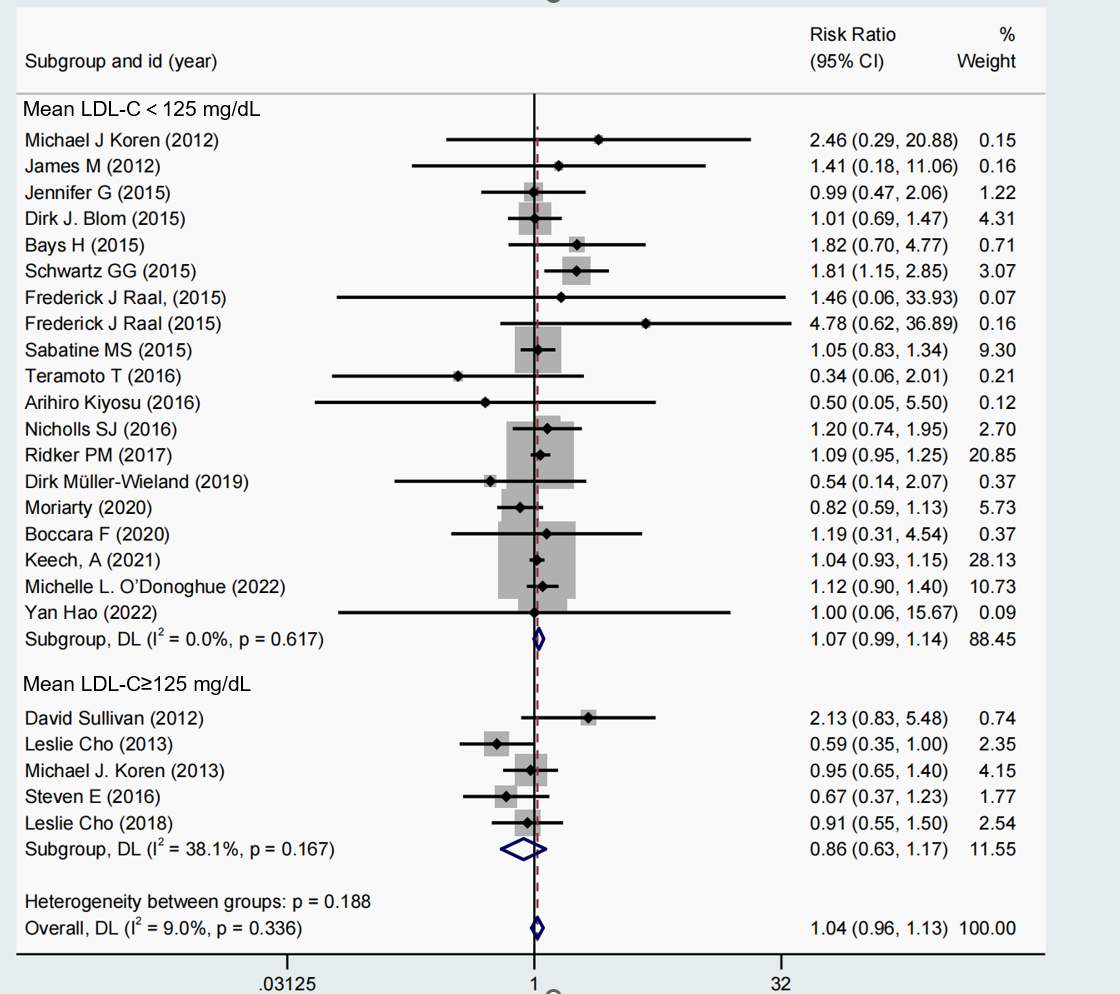


**Supplementary Figure 6A. Forest plot for Mean LDL-C.The risk of New Muscle Symptom of PCSK9i and Ezetimibe in hyperlipidemic patients.**


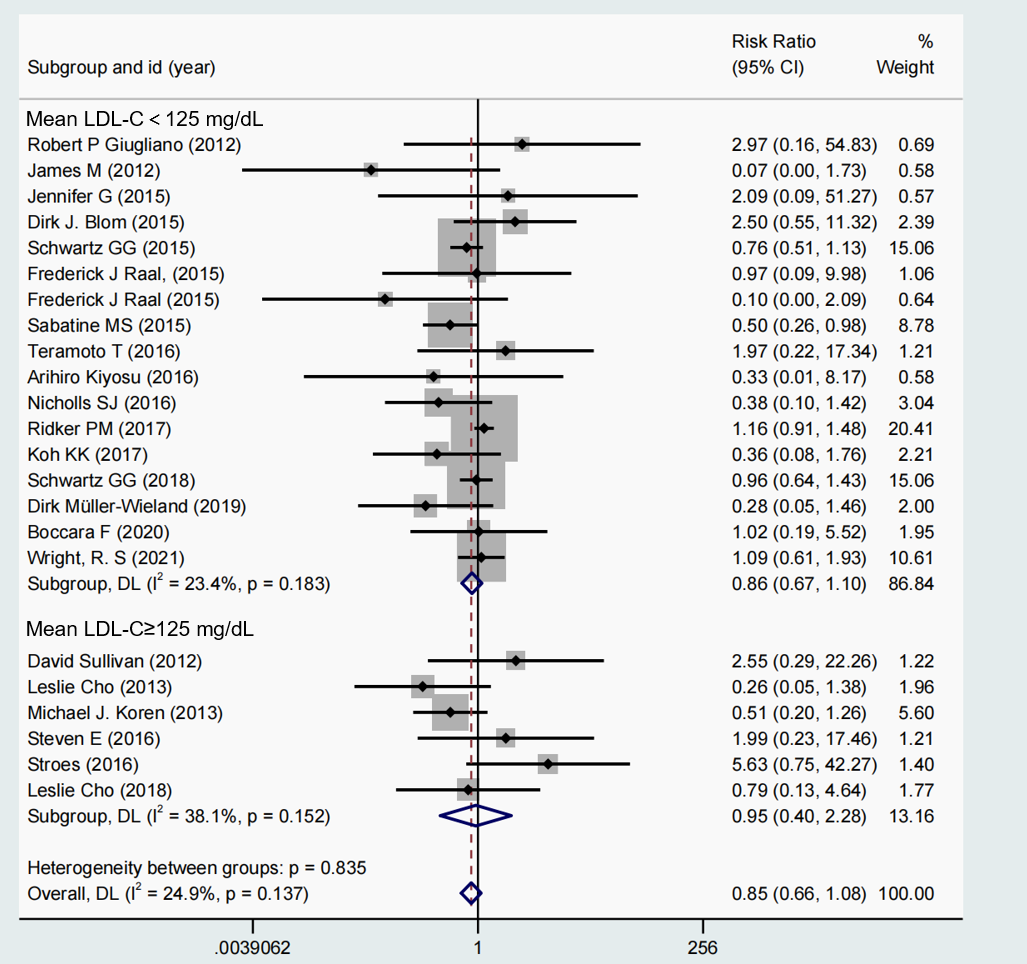


**Supplementary Figure 6B. Forest plot for mean Mean LDL-C.The The risk of Creatine Kinase >3ULN of PCSK9i and Ezetimibe in hyperlipidemic patients.**
